# Crisis communication in the WHO COVID-19 press conferences: A retrospective analysis

**DOI:** 10.1371/journal.pone.0282855

**Published:** 2023-03-13

**Authors:** Sike He, Dapeng Li, Chang-Hai Liu, Ying Xiong, Dan Liu, Jiaming Feng, Ju Wen

**Affiliations:** 1 West China School of Medicine, Sichuan University, Chengdu, Sichuan, China; 2 West China School of Pharmacy, Sichuan University, Chengdu, Sichuan, China; 3 Center of Infectious Diseases, West China Hospital, Sichuan University, Chengdu, Sichuan, China; 4 Department of Periodical Press/Chinese Evidence-based Medicine Center, West China Hospital, Sichuan University, Chengdu, Sichuan, China; 5 Department of Periodical Press, West China Hospital, Sichuan University, Chengdu, Sichuan, China; 6 School of Liberal Education, Chengdu Jincheng College, Chengdu, Sichuan, China; Sapienza University of Rome, ITALY

## Abstract

**Objectives:**

The objective of this study is to investigate, from a longitudinal perspective, how WHO communicated COVID-19 related information to the public through its press conferences during the first two years of the pandemic.

**Methods:**

The transcripts of 195 WHO COVID-19 press conferences held between January 22, 2020 and February 23, 2022 were collected. All transcripts were syntactically parsed to extract highly frequent noun chunks that were potential topics of the press conferences. First-order autoregression models were fit to identify “hot” and “cold” topics. In addition, sentiments and emotions expressed in the transcripts were analyzed using lexicon-based sentiment/emotion analyses. Mann-Kendall tests were performed to capture the possible trends of sentiments and emotions over time.

**Results:**

First, eleven “hot” topics were identified. These topics were pertinent to anti-pandemic measures, disease surveillance and development, and vaccine-related issues. Second, no significant trend was captured in sentiments. Last, significant downward trends were found in anticipation, surprise, anger, disgust, and fear. However, no significant trends were found in joy, trust, and sadness.

**Conclusions:**

This retrospective study provided new empirical evidence on how WHO communicated issues pertaining to COVID-19 to the general public through its press conferences. With the help of the study, members of the general public, health organizations, and other stake-holders will be able to better understand the way in which WHO has responded to various critical events during the first two years of the pandemic.

## Introduction

The outbreak of coronavirus disease 2019 (COVID-19) is an unprecedented public health crisis that has posed enormous challenges to the global healthcare systems and the general public [1,2]. These challenges range from the psychological maladjustment to the widespread misinformation on social media, and the confusion pertaining COVID-19 vaccines. In face of these challenges, timely and effective communication is needed to promote information transparency, which in turn, helps keep members of the public informed of the pandemic [1,3]. Therefore, understanding how information is communicated to facilitate the prevention and control of COVID-19 is a key research question.

The research on crisis communication during the COVID-19 pandemic has attracted extensive attention from the research community [4–8]. One line of research focuses on the sentiments or emotions expressed on social media. For example, Li et al. [9] examined changes in emotions and sentiments before and after the declaration of COVID-19 in China on the social media platform Weibo. It was found that negative emotions and sensitivity to social risks increased whereas positive emotions and life satisfaction decreased after the declaration. In addition, the public focused more on health and family than leisure and friends. Similarly, Xue et al. [10] examined the public discourse and emotions related to COVID-19 on Twitter. They found that fear arose when new confirmed cases or deaths were reported.

Another line of research examines the official announcements and statements on COVID-19 made by a local or national government [1,3,11–13]. For example, Antiwi and Nyarkoh [12] examined official statements released by the Ghana government during the COVID-19 pandemic. They found that the government always tried to spread calmness, rather than fear, to help build trust and confidence. Similarly, Schueler and Marx [14] analyzed the speech act in COVID-19 press conferences from the Dutch government. They found that assertive statements were more frequently used in neutral press conferences than in easing or tightening press conferences. Thus, the focus of neutral press conferences was to inform people on the current state of affairs. In addition, emotions of press conferences were closely associated with the development of the pandemic. That is, emotions became more tightening when facing a shortage of medical resources while emotions would be easing when situations were improved.

The aforementioned studies have contributed significantly to our understanding of crisis communication in the pandemic. However, these findings seemed inconclusive. On the one hand, most of these studies focused on opinions expressed by individuals or officials who are not medical professionals. Since the control and prevention of COVID-19 requires highly specialized knowledge in medical sciences, messages from medical professionals or public health institutions are of particular importance. On the other hand, as one of the top public health organizations, the World Health Organization (WHO) plays an critical role in supporting countries to prepare for, respond to, and recover from the pandemic [15]. The WHO COVID-19 press conference is one of the most important channels for WHO to communicate with the public during the pandemic. The press conferences covered all important issues pertaining to COVID-19 and were widely reported by international media. Despite the immense influence, how did WHO use the press conferences as a means to coordinate its responses to the prevention and control of disease and inform the public remains largely unknown.

The objective of this study is to understand, from a longitudinal perspective, how WHO communicated COVID-19 related information through its press conferences during the first two years of the pandemic. To achieve that objective, we set out to investigate the topics, the expression of sentiments and emotions [16] in the WHO COVID-19 press conferences. The following research questions guided the study.

RQ1: What were “hot” and “cold” topics in WHO COVID-19 press conferences during the first two years of the pandemic?

RQ2: How were sentiments and emotions expressed in WHO COVID-19 press conferences evolve during the first two years of the pandemic?

## Methods

### Data collection

The dataset used in this study was a corpus of WHO COVID-19 press conferences transcripts curated in the first week of March, 2022. We manually downloaded transcripts of the press conferences held between January 22, 2020 and February 23, 2022 from the official website of WHO. Since March 2, 2022, the press conferences covered both coronavirus and different events (e.g., the war in Ukraine). As the focus of the present study was coronavirus, transcripts released after that date were not included in the corpus. In other words, we have collected all the available samples of the press conference transcripts at the time of data collection. Because we aimed to trace the possible diachronic change of topics, sentiments, and emotions by month, all transcripts in each month were merged into one text file (see Table 1).

**Table 1:** Descriptive statistics of the WHO COVID-19 press conference corpus.

	Date	Number of transcripts	Word count	Sentence count	Mean text length	Mean sentence count
1	2020–01	4	25,203	1,419	6,300.750	354.750
2	2020–02	18	118,194	5,811	6,566.333	322.833
3	2020–03	14	107,350	5,374	7,667.857	383.857
4	2020–04	13	109,578	5,183	8,429.077	398.692
5	2020–05	12	104,444	4,731	8,703.667	394.250
6	2020–06	13	100,317	4,532	7,716.692	348.615
7	2020–07	9	67,299	3,135	7,477.667	348.333
8	2020–08	9	73,824	3,660	8,202.667	406.667
9	2020–09	9	81,796	3,970	9,088.444	441.111
10	2020–10	8	70,696	3,250	8,837.000	406.250
11	2020–11	7	62,631	2,715	8,947.286	387.857
12	2020–12	7	66,472	3,003	9,496.000	429.000
13	2021–01	7	74,272	3,291	10,610.286	470.143
14	2021–02	8	63,576	2,965	7,947.000	370.625
15	2021–03	8	72,049	3,172	9,006.125	396.500
16	2021–04	9	74,305	3,670	8,256.111	407.778
17	2021–05	6	45,222	2,099	7,537.000	349.833
18	2021–06	6	43,270	2,342	7,211.667	390.333
19	2021–07	5	39,004	1,683	7,800.800	336.600
20	2021–08	4	31,248	1,369	7,812.000	342.250
21	2021–09	2	15,336	723	7,668.000	361.500
22	2021–10	4	27,671	1,419	6,917.750	354.750
23	2021–11	3	23,518	1,110	7,839.333	370.000
24	2021–12	5	52,045	2,442	10,409.000	488.400
25	2022–01	3	24,577	1,111	8,192.333	370.333
26	2022–02	2	14,235	689	7,117.500	344.500
	Total	195	1,588,132	74,868	8,144.267	383.938

### Topic extraction

First, all transcripts in the dataset were lower-cased and lemmatized. An initial close reading on the original transcript texts revealed two types of inconsistencies in spelling (e.g., ‘covid—19’ versus ‘covid-19’, and ‘lock down’ versus ‘lock-down’). To resolve the differences, all occurrences of ‘covid—19’ and ‘lock down’ were replaced with ‘covid-19’ and ‘lock-down’ respectively.

Second, following the methods proposed in [17], the open-source Natural Language Processing (NLP) library *spacy* [18] was used to parse the syntactic dependency relations [19] of each sentence in the transcripts. Then, all noun chunks, together with their frequencies and range counts, were automatically extracted from the parsed results.

Third, the parsed results were further filtered based on the repetition criteria (i.e., frequency and range) [17,20,21]. After several rounds of experiment, the final threshold of the repetition criteria was set at 16 for both frequency and range. That is, a noun chunk was considered as a potential topic if it occurred at least 16 times in the entire parsed dataset from at least 16 different transcripts. The repetition criteria were set based on the consideration of frequency and practicality. To be specific, a noun chunk should be deemed as a candidate topic when it had frequent occurrence and spread across a wide range of transcripts. More importantly, the size of the candidate noun chunks should be manageable. Noun chunks that failed to meet the repetition criteria were removed. Two researchers independently checked each of the remaining noun chunks to decide whether a noun chunk was a possible topic. When discrepancy occurred, the researchers discussed until a full agreement was reached. Note that only noun chunks that were considered as candidate topics were kept for further analyses.

Last, the normalized frequency of each noun chunk by month was computed using Formula 1 to make the comparison of noun chunks across each month more legitimate.


$$Normalized\;Frequency\;\left(Noun\;Chunk\right)=\frac{Raw\;Frequency}{Number\;of\;Press\;Briefings\;of\;a\;Month}$$ (1)

### Sentiment and emotion analyses

Lexicon-based sentiment and emotion analyses were performed on transcripts with the R package *sentimentr* [22,23]. For sentiment analysis, the *sentimentr* package utilizes the Jockers-Rinker sentiment lexicon [24] to determine whether the overall opinion expressed in a transcript was positive (sentiment score > 0), negative (sentiment score < 0), or neutral (sentiment score = 0). For emotion analysis, the *sentimentr* package relies on the NRC emotion lexicon [25] to compute scores of eight categories emotions in Plutchik’s model of emotions [26] which includes anger, anticipation, disgust, fear, joy, sadness, surprise, and trust.

Note that although both sentiment analysis and emotion analysis are concerned with the affective dimension of human language, they work at different granularity and are designed to gauge different psycholinguistic features [27]. In addition, since sentiment and emotion analyses were conducted separately in the present study, using different lexicons would not have impact on the results.

### Statistical analyses

Different from other topic extraction in NLP which automatically generate a given number of topics by clustering a group of key words based on machine-learning techniques, the current topic extraction process relied on the calculating the frequency and range of noun chunks (i.e., topics). Since we are interested in how topics evolved in the time frame examined, a statistical technique which can relate a variable to its past values in the time series was needed. Similarly, a technique that is able to capture to the trends of sentiments and emotions over time is also needed to serve our research goal.

For RQ1, first-order autoregression models [21,28] were fit to identify “hot” and “cold” topics based on the normalized frequency of noun chunks in each month. A noun chunk which was detected with a significant increase of normalized frequencies was considered a “hot” topic, whereas a significant decrease was indicative of a “cold” topic [24].

For RQ2, Mann-Kendall Trend tests were performed to detect possible trends in the overall sentiment and eight categories of emotions over time. Sen’s slope and the corresponding confidence intervals were computed for the rate of change.

## Results

### Topics

A total of 91 topics were identified in the dataset (see S1 Table). The top 10 topic in terms of raw frequencies were *health system*, *severe disease*, *act accelerator*, *covax facility*, *contact tracing*, *healthcare worker*, *public health*, *young people*, *clinical trial*, and *physical distancing*.

**Fig 1:**
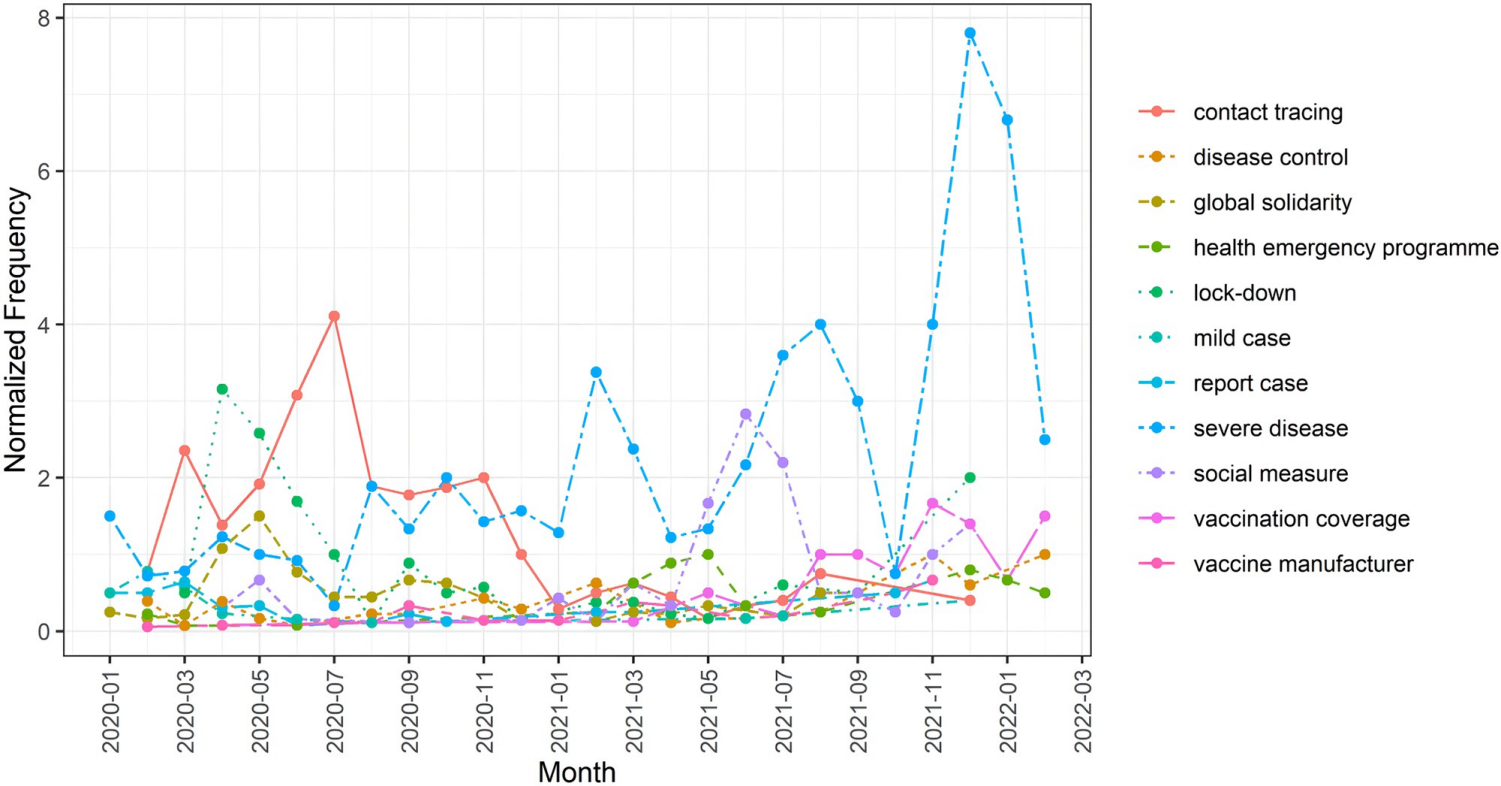
Trajectory of hot topics between January, 2020 and February, 2022.

Results of the first-order autoregressive models showed that, of the 91 topics, the normed frequencies of 11 topics increased significantly over the past 26 months (see Table 2 and Fig 1). These topics were considered ‘hot’ topics. Interestingly, none of the topics showed significant decreasing trend in the period. That is, no significant cold topics were identified.

**Table 2:** Hot topics in the WHO COVID-19 press conferences.

Topic	Frequency	Dispersion	AR coefficient	*p*-value
vaccine manufacturer	24	20	0.755	0.01
contact tracing	259	104	0.672	<0.001
report case	41	30	0.653	0.003
mild case	35	22	0.649	0.003
severe disease	345	124	0.581	<0.001
vaccination coverage	35	25	0.566	0.024
social measure	83	45	0.562	0.002
global solidarity	83	51	0.55	0.002
health emergency programme	38	34	0.506	0.026
disease control	44	34	0.489	0.035
lock-down	151	62	0.481	0.024

### Sentiments and emotions

**Fig 2:**
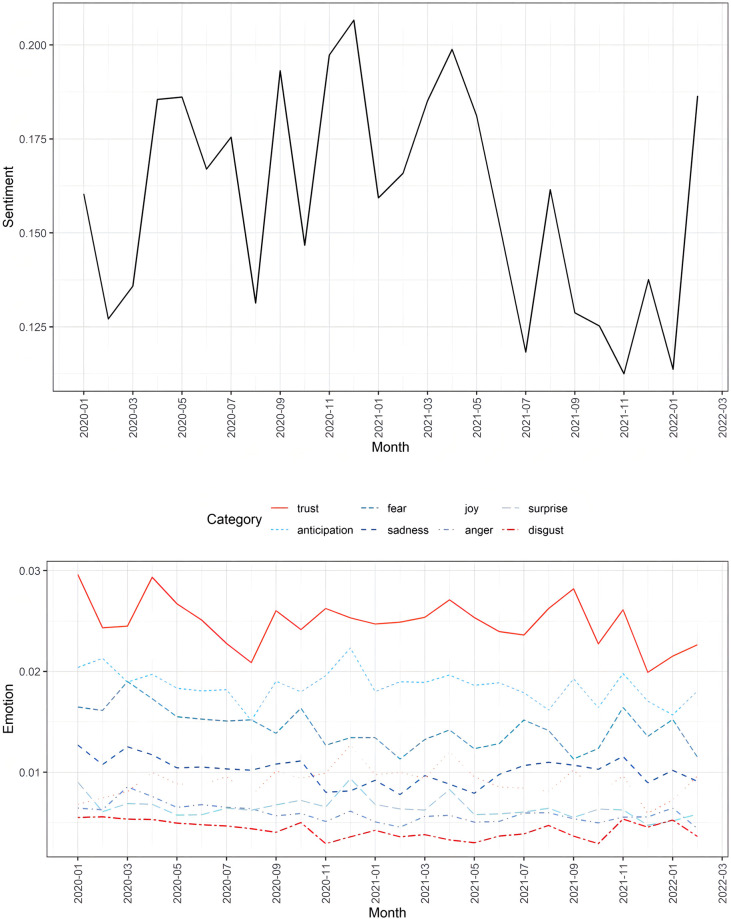
Sentiment and emotion trajectory of WHO COVID-19 press conferences.

Results of the Mann-Kendall trend test on sentiment scores (Z = -1.2343, τ = -0.1753846, *p*. = 0.2171) showed that no significant trend in sentiment was found between January, 2020 and February, 2022. Although the overall sentiment was positive, clear fluctuations were observed during the period (see Fig 2A). That is, the trajectory of sentiment underwent constant changes as the pandemic developed.

Results of the emotion analysis showed that trust and anticipation were the most predominant categories of emotion, followed by fear, sadness, joy, surprise, anger, and disgust (see Fig 2B). In addition, results of the Mann-Kendall trend tests indicated that significant downward trends were found in anticipation, surprise, anger, disgust, and fear. However, no significant trends were found in joy, trust, and sadness (see Table 3).

**Table 3:** Statistics of the Mann-Kendall trend tests on emotions.

Emotion	*n*	*Z*	*p*-value	*τ*	Sen’s slope	Trend
anticipation	26	-2.072	0.038	-0.292	-8.22062e-05	Downtrend
joy	26	0.044	0.965	0.009	1.410294e-06	No
trust	26	-1.543	0.123	-0.218	-0.000122036	No
surprise	26	-2.336	0.019	-0.329	-5.157525e-05	Downtrend
anger	26	-3.218	0.001	-0.452	-6.946889e-05	Downtrend
disgust	26	-2.557	0.011	-0.360	-7.208925e-05	Downtrend
fear	26	-2.998	0.003	-0.422	-0.000180047	Downtrend
sadness	26	-1.851	0.064	-0.262	-7.080847e-05	No

## Discussion

The present study investigated the topics, sentiments, and emotions in the WHO COVID-19 press conferences. Results of our study showed that 1) the normalized frequencies of 11 topics (noun chunks) increased in the period, 2) no significant monotonic trend in sentiment was found, and 3) trust, anticipation, and fear were the most predominate categories of emotions. Below, we discuss the findings in details with possible explanations.

A close reading revealed that these “hot topics” fall into three broad categories: 1) anti-pandemic measures (i.e., contact tracing, lock-down, global emergency programme, disease control, and global solidarity); 2) disease surveillance and development (i.e., mild case, report case, and severe disease); 3) vaccine-related issues (i.e., vaccination coverage, and vaccination manufacturer). Topics pertaining to anti-pandemic measures (e.g., contact tracing) attracted public attention mainly in the early stage of the pandemic [10,29,30]. On January 30 of 2020, the WHO officially declared the outbreak of COVID-19 a public health emergency concern. Since then, it has been suggested that the transmission of COVID-19 can be blocked by measures such as contact tracing systems, isolation and lock-down [31,32]. Topics concerning disease surveillance and development were stable in terms of frequency except the “severe disease”. The term “severe disease” has attracted increasing attention probably because it is associated with the rate of mortality [10,29,30,33]. In addition, severe disease is also important for studying the treatment, prognosis, control measures of the disease [31,34], especially when new variants were detected (e.g., Delta, Omicron) [35–38]. As vaccine has been considered one of the key solutions to the pandemic and multiple pharmaceutical companies and institutions (i.e., vaccination manufacturer) around the globe have actively committed to developing COVID-19 vaccines [5,39].

Contrary to the sentiment of COVID-19 related messages detected in social media [9,10,40,41], no significant trend of sentiment was observed in the WHO COVID-19 press conferences. Although the official statements are usually presented in an objective and calm manner, Frewer et al. [42] argued that if government or institutions failed to respond to a crisis timely, public outrage and instability may arise. In this connection, fluctuations in the sentiment may be observed as significant events occurred. For example, the rises of sentiment in September and November in 2020 were seemingly corroborated with the major progress in vaccine development at that time. Since vaccines are crucial in fighting the pandemic, these breakthroughs would contribute to the positive sentiments [5]. In contrast, the decrease of sentiment may be associated with negative events such as virus mutation or the severe situations during the pandemic. For example, the values of sentiment scored relatively low when the Delta variant was detected in October 2020 [35,43] and the Omicron variant was detected in November 2021[37,38]. In February 2020, the number of confirmed cases and death cases rose sharply [31], which in turn, triggered the negative sentiment.

In line with previous studies of emotions on COVID-19 [9,10,44,45], our findings suggested that anticipation and trust were the most predominate emotions. These two emotions are more inclined to reflect group cohesiveness instead of individual emotions [9]. Tziner [46] argued that group threats such as natural disasters and pandemic can make groups a community of interests and generate more beneficial and positive behaviors and social solidarity. Under such conditions, the prevalence of positive psychological reactions such as trust and anticipation can enhance public’s coping ability [47,48] and increase trust in government during a crisis [49,50]. The emotions “trust” and “anticipation” can be generated by some situation improvement such as the decline in the number of people involved in crisis (i.e., confirmed and death cases) and government’s effective effort [51,52]. As the most authoritative health organization, WHO needs to spread trust and claim to help build confidence in fighting COVID-19 [53]. On the other hand, previous studies also suggest that public health emergencies can trigger a series of negative emotional responses, including a higher level of anxiety, anger, depression and fear [9,54–56]. For example, “fear” is usually related to emergencies and crisis, since it is one of the most pivotal emotions linked with life safety and survival [57,58]. Although these emotions also can be captured in our result, the levels were low indicating WHO’s effort to avoid spreading negative emotions. In other words, WHO has consistently been positive in tone regardless of the status quo of the pandemic, which in turn, may have helped the public build confidence and look the bright side of life. In terms of the trends of emotions, positive psychological reactions (i.e., joy, anticipation, trust, surprise) exhibited a downward trend while negative emotions such as “fear” exhibited an increasing trend during the early stage of COVID-19 pandemic. Then with the growing number of recovered patients and the development of vaccines, the trends of trust and joy turned upward [5,51].

## Conclusions

The retrospective study provided new empirical evidence on how WHO communicated issues pertaining to COVID-19 to the general public through its press conferences. With the help of this research, members of general public, health organizations, and other stake-holders will be able to better understand the way in which WHO has responded to various critical events during the first two years of the pandemic.

Since we only examined the monotonic trend, which may not be sufficient to account for the dynamic development of sentiments and emotions over the period. Future studies may consider examine these trends with different measures.

## Supporting information

S1 Table Topics in WHO COVID-19 press conferences from January, 2020 to February, 2022. (DOCX)
