# Supplementary material for: Crisis communication in the WHO COVID-19 press conferences: A retrospective analysis
Source: PLoS One. 2023 Mar 13;18(3):e0282855. doi: 10.1371/journal.pone.0282855 (PMC10010532; doi:10.1371/journal.pone.0282855)
Supplement: S1 Table — (DOCX) [file pone.0282855.s001.docx]

**Appendix 1**

Table S1. Topics in WHO COVID-19 press conferences from January, 2020 to February, 2022

| Topic | Frequency | Dispersion | AR(1) coefficient | *p-*value |
| --- | --- | --- | --- | --- |
| act accelerator | 340 | 95 | -0.025 | 0.906 |
| acute phase | 39 | 21 | 0.48 | 0.109 |
| astrazeneca vaccine | 85 | 27 | 0.469 | 0.093 |
| asymptomatic infection | 20 | 17 | -0.271 | 0.378 |
| booster dose | 64 | 22 | 0.351 | 0.186 |
| climate change | 45 | 18 | -0.053 | 0.835 |
| clinical care | 60 | 47 | -0.121 | 0.603 |
| clinical trial | 194 | 85 | 0.115 | 0.582 |
| close contact | 58 | 38 | -0.417 | 0.339 |
| community transmission | 137 | 69 | -0.034 | 0.88 |
| comprehensive approach | 97 | 55 | -0.164 | 0.431 |
| contact tracing | 259 | 104 | 0.672 | <0.001 |
| covax facility | 286 | 70 | 0.289 | 0.211 |
| covid- pandemic | 140 | 84 | 0.196 | 0.337 |
| covid- vaccine | 105 | 59 | 0.295 | 0.166 |
| crowded space | 43 | 33 | -0.068 | 0.791 |
| death rate | 30 | 23 | -0.235 | 0.362 |
| delta variant | 120 | 24 | 0.546 | 0.069 |
| develop country | 74 | 46 | -0.121 | 0.589 |
| disease control | 44 | 34 | 0.489 | 0.035 |
| disease transmission | 18 | 18 | 0.115 | 0.756 |
| emergency response | 26 | 22 | 0.043 | 0.885 |
| equitable access | 88 | 45 | -0.108 | 0.636 |
| essential health service | 47 | 34 | 0.217 | 0.418 |
| fatality rate | 24 | 23 | 0.363 | 0.439 |
| first wave | 35 | 24 | 0.072 | 0.809 |
| full genome sequence | 30 | 23 | -0.193 | 0.461 |
| global level | 80 | 52 | 0.407 | 0.066 |
| global solidarity | 83 | 51 | 0.55 | 0.002 |
| hand hygiene | 82 | 57 | -0.026 | 0.908 |
| health emergency | 36 | 30 | 0.151 | 0.636 |
| health emergency programme | 38 | 34 | 0.506 | 0.026 |
| health facility | 72 | 49 | -0.267 | 0.217 |
| health service | 56 | 31 | 0.19 | 0.451 |
| health system | 353 | 134 | 0.167 | 0.426 |
| healthcare facility | 53 | 33 | 0.091 | 0.763 |
| healthcare system | 63 | 46 | -0.289 | 0.208 |
| healthcare worker | 230 | 85 | 0.193 | 0.337 |
| herd immunity | 61 | 25 | -0.072 | 0.76 |
| high-income country | 71 | 42 | 0.183 | 0.408 |
| immune response | 66 | 34 | 0.039 | 0.916 |
| infection prevention | 43 | 35 | 0.004 | 0.988 |
| infectious disease | 59 | 42 | -0.237 | 0.309 |
| intense transmission | 33 | 24 | -0.41 | 0.428 |
| intensive care | 39 | 30 | 0.277 | 0.301 |
| lock-down | 151 | 62 | 0.481 | 0.024 |
| low and middle-income country | 76 | 41 | -0.106 | 0.745 |
| manufacturing capacity | 29 | 18 | 0.311 | 0.223 |
| mass gathering | 55 | 29 | -0.035 | 0.889 |
| mental health | 64 | 30 | -0.102 | 0.663 |
| mild case | 35 | 22 | 0.649 | 0.003 |
| mrna vaccine | 29 | 20 | -0.299 | 0.651 |
| new vaccine | 48 | 33 | 0.059 | 0.807 |
| new variant | 101 | 42 | 0.046 | 0.88 |
| new virus | 57 | 38 | 0.271 | 0.454 |
| old people | 107 | 58 | 0.053 | 0.807 |
| pcr test | 43 | 26 | 0.172 | 0.484 |
| personal protective equipment | 67 | 55 | 0.296 | 0.165 |
| pfizer vaccine | 48 | 20 | 0.125 | 0.665 |
| physical distance | 35 | 26 | 0.344 | 0.155 |
| physical distancing | 186 | 98 | 0.085 | 0.716 |
| primary healthcare | 36 | 23 | -0.292 | 0.252 |
| private sector | 135 | 53 | -0.111 | 0.604 |
| public health | 222 | 107 | 0.331 | 0.071 |
| public health and social measure | 45 | 33 | -0.218 | 0.393 |
| public health emergency | 56 | 32 | 0.14 | 0.866 |
| public health measure | 117 | 68 | 0.132 | 0.525 |
| public health system | 20 | 16 | 0.362 | 0.199 |
| report case | 41 | 30 | 0.653 | 0.003 |
| respiratory disease | 26 | 21 | 0.112 | 0.782 |
| respiratory etiquette | 41 | 37 | 0.002 | 0.994 |
| respiratory pathogen | 24 | 17 | -0.311 | 0.416 |
| rich country | 38 | 26 | 0.125 | 0.613 |
| safe and effective vaccine | 46 | 36 | 0.286 | 0.238 |
| severe disease | 345 | 124 | 0.581 | <0.001 |
| social distancing | 53 | 31 | -0.183 | 0.442 |
| social measure | 83 | 45 | 0.562 | 0.002 |
| solidarity trial | 109 | 41 | 0.127 | 0.703 |
| supply chain | 61 | 43 | 0.117 | 0.612 |
| surveillance system | 30 | 27 | -0.295 | 0.249 |
| suspect case | 95 | 42 | 0.175 | 0.528 |
| universal health coverage | 47 | 34 | 0.004 | 0.987 |
| vaccination coverage | 35 | 25 | 0.566 | 0.024 |
| vaccine development | 42 | 29 | 0.137 | 0.584 |
| vaccine equity | 75 | 30 | -0.071 | 0.817 |
| vaccine manufacturer | 24 | 20 | 0.755 | 0.001 |
| vaccine supply | 25 | 18 | 0.097 | 0.735 |
| vaccine trial | 26 | 17 | 0.292 | 0.382 |
| virus variant | 63 | 31 | -0.252 | 0.468 |
| vulnerable people | 32 | 26 | 0.186 | 0.425 |
| young people | 210 | 43 | -0.219 | 0.333 |
